# Study of the Electrical Conductivity of Ion-Exchange Resins and Membranes in Equilibrium Solutions of Inorganic Electrolytes

**DOI:** 10.3390/membranes12020243

**Published:** 2022-02-20

**Authors:** Oleksandr Petrov, Natalia Iwaszczuk, Irina Bejanidze, Tina Kharebava, Volodymyr Pohrebennyk, Nato Didmanidze, Nunu Nakashidze

**Affiliations:** 1 Faculty of Management, AGH University of Science and Technology, Mickiewicza Av. 30, 30-059 Krakow, Poland; niwaszcz@zarz.agh.edu.pl; 2 Faculty of Natural Sciences and Health Care, Department of Chemistry, Batumi Shota Rustaveli State University, Ninoshvili/Rustaveli Str. 35/32, Batumi 6010, Georgia; irina.bejanidze@bsu.edu.ge (I.B.); tina.kharebava@bsu.edu.ge (T.K.); nato.didmanidze@bsu.edu.ge (N.D.); 3 Department of Ecological Safety and Nature Protection Activity, V. Chornovil Institute of Sustainable Development, Lviv Polytechnic National University, Bandery Str., 12, 79013 Lviv, Ukraine; volodymyr.d.pohrebennyk@lpnu.ua; 4 Department of Agroecology and Forestry, Batumi Shota Rustaveli State University, Batumi 6010, Georgia; nunu.nakashidze@bsu.edu.ge

**Keywords:** electrical conductivity, ion-exchange membranes, ion exchange resins

## Abstract

The study of the electrical conductivity of ion-exchange membranes in equilibrium electrolyte solutions is of great importance for the theory of membrane processes, in particular for practical electrodialysis. The purpose of the work is to determine the electrical conductivity of industrial ion-exchange membranes MK-40 and MA-40, as well as their basis—granules of a bulk layer of industrial ion exchangers KU-2-8 and EDE-10p, by differential and modified contact methods in electrolyte solutions and the development of a new methodology that will give the values that are closest to the true ones; determination of the dependence of electrical membrane conductivity depending on the type of counterion and concentration equilibrium solution and granules of a bulk layer of ion exchangers on the volume fraction of a dry ion exchanger with different degrees of compaction. It is shown that the dependence of the electrical conductivity of diaphragms on the electrolyte concentration, according to theoretical ideas, disappears under compression. It has been experimentally established that the difference method gives lower values of electrical conductivity in the region of low concentrations. The data obtained by the contact method are in good agreement with the results obtained for compressed diaphragms. The membrane conductivity decreases with increasing ion size.

## 1. Introduction

Electrodialysis is one of the methods of membrane technology for the separation and purification of liquids [1,2,3,4,5]. According to the forecasts of the development of the world economy, membrane technology is regarded as the technology of the future [6,7,8,9,10]. It has been widely used in many industries and agriculture. Membrane processes are used in almost all spheres of human activity, since they ensure high efficiency of processes, provide the population with drinking water, protect the environment, etc., [11,12,13,14,15]. Electrodialysis is an environmentally friendly process with low energy consumption [16,17,18,19].

The application of this method allows you to successfully carry out the processes of desalination and concentration of solutions, obtaining drinking water from saltwater, treatment of natural and industrial wastewaters, etc. It is widely introduced in the energy, electronic, chemical and food industries, in medicine, agriculture and other spheres of human activities [10,20,21,22,23,24].

Ion-exchange membranes are used in electrodialysis for the concentration or desalting of aqueous or non-aqueous electrolytic systems, most often containing not only inorganic substances but also organic substances [10], and in diffusion dialysis, to extract acids or alkalis from spent acid or alkaline solutions [13]. In addition, ion-exchange membranes are largely used in membrane fuel cells, flow batteries [16,17], reverse electrodialysis [18,19], electrolyzers [20], microfluidic devices, chemical synthesis [22] and others.

In synthetic ion exchangers, there are always counterions next to the fixed ions of the matrix, which compensate for their charge and determine the ability of the ion-exchange membranes to pass an electric current. The concentration of both types of ions is quite high. When immersed in a solution, mobile counterions are able to exchange for electrolyte ions in the solution. In addition, the solvent and some additional amount of counterions are absorbed, together with which, due to the preservation of the electroneutrality of the system, an equivalent amount of ions with the same charge as the matrix penetrates. The additionally absorbed electrolyte is an unchangeably absorbed or Donnan-sorbed electrolyte. When a constant electric field is applied to the membrane, the lines of force that are oriented perpendicular to the surface of the membrane will pass only counterions through itself, not letting co-ions pass. This property of ion-exchange membranes underlies their widespread use for separating substances in electrodialyzers [25,26].

The selectivity of an ion-exchange membrane is the number of transfer of counterions, which for an ideally selective membrane is equal to 1, and for real membranes, it ranges from 0.94 to 0.98 (in 0.1 N NaCl). High selectivity is manifested in ion-exchange membranes with high electrochemical activity (specific electrical conductivity), low hydrodynamic, diffusion, and osmotic permeability, i.e., primarily in membranes of a regular structure with a large number of highly ionized functional groups. Ion-exchange membranes based on strongly and weakly ionized cation exchangers and anion exchangers find the greatest application in the technology of water desalination [27,28,29,30,31].

Some of the most important properties of ion-exchange membranes are conductive properties, such as electrical conductivity, selectivity, transfer numbers, diffusion permeability of membranes, and others. There is no unified theory or perfect model for describing the dependence of the electrical conductivity of membranes on the concentration of an equilibrium solution. Therefore, the experimental determination of the specific conductivity of ion-exchange membranes in equilibrium solutions of various concentrations (C) is of primary importance for the theory of membrane processes, the practice of electrodialysis and other methods of membrane separation [32,33,34,35].

### Electrical Conductivity of Ion-Exchange Membranes and Comparison of Methods for Its Determination

Electrical conductivity is the ability to conduct electrical current. The electrical conductivity of the membrane depends on the concentration of fixed charges in it, i.e., on its exchange capacity. The electrical conductivity of the membrane is also affected by the degree of interaction between fixed charges and counterions, which sometimes results in the formation of ion pairs that firmly bind counterions, and hence a decrease in the number of counterions in the membrane. The physical structure of the membrane (homogeneity, degree of porosity) has a significant effect on electrical conductivity. Ion-exchange membranes are capable of passing an electric current, which implies the presence of mobile ions in the membrane phase. Since these are only counterions of the membrane and the external solution, when a constant electric field is applied to the membrane, the lines of force that are oriented perpendicular to the surface of the membrane will only pass counterions, not letting the co-ions pass. This property of ion-exchange membranes is the basis for their widespread use for the separation of substances in electrodialyzers. Distinguishing between specific and molar electrical conductivity.

Methods for measuring the electrical conductivity of membranes (ǣ_m_) are divided into two large groups: methods for measuring conductivity in the longitudinal and transverse directions. The methods for measuring the longitudinal conductivity of membranes are relatively simple, but they all give an overestimated value of the electrical conductivity (especially in the region of concentrated solutions) due to the surface conductivity of the solution film. In addition, knowledge of the conductivity of the membrane in the transverse direction is of greater practical value than in the longitudinal direction. Therefore, methods for measuring the transverse electrical conductivity of membranes are more widely used. Among the methods for measuring the transverse electrical conductivity of membranes, there are two main groups: contact and difference methods. The main advantages of contact methods are the rapidity of a single measurement and the simplicity of the hardware design.

In contact measurement methods ǣ_m_ (mercury, pressure contacts, probes and agar bridges supplied to the membrane), the membrane–electrode contact resistance is included in the measured electrical resistance, while the researchers cannot avoid the passage of current through the interlayers and hydrophilic films of the solution [36,37,38,39,40,41,42]. This leads to a significant increase in resistance even with a negligible membrane thickness and especially in dilute solutions with specific electrical conductivity æ_v_ << ǣ_m_, to a decrease in ǣ_m_. Along with this, the values of ǣ_m_ are distorted due to the polarization of the electrodes, drying out and deformation of the membranes.

To measure the resistance of membranes by the contact method, it is sufficient to clamp the test sample between flat metal electrodes so that there is membrane–electrode contact over the entire surface of their contact. Of the many designs of contact-type cells, one should note the design proposed by Larchet [43] in the form of a clothespin, at one end of which platinized platinum electrodes are fixed parallel to each other. The constant pressure on the membrane clamped between the electrodes is ensured by a tightening rubber band. The cell is convenient in that it allows hundreds of measurements per day to be performed with an accuracy sufficient for a comparative analysis of the electrical conductivity of different membranes.

It is possible to avoid membrane deformation during measurements by the contact method by using mercury electrodes. For example, it was found by the mercury contact method that the specific electrical conductivity of heterogeneous membranes is about an order of magnitude lower than that of the corresponding ion exchangers. The electrical conductivity of MK-40 and MA-40 membranes in 0.1 N. NaCl at 20 °C, measured by the mercury-contact method, is 4.49 × 10^−3^ and 5.15 × 10^−3^ S/cm, respectively. An increase in the temperature of the solution to 60 °C leads to an increase in electrical conductivity by an average of 2.5–3 times, that is, by 1.5–2% per degree, which is explained by an increase in the mobility of ions. The electrical conductivity also increases with an increase in the concentration of the solution. Determination of the resistance of membranes to alternating current by the contact method is complicated by an additional contribution to the measured value of the resistances of the membrane–electrode boundaries, leading to an overestimation of the sought resistance of the membranes and making it frequency-dependent. The membrane/mercury transition resistance can be taken into account by measuring the frequency dependence of the active component of the cell impedance. Since in all modifications of the contact method of the transverse resistance of membranes, the contribution of the transition boundaries, electrode–membrane or membrane–membrane, to the value of the measured resistance, the method gives underestimated values of electrical conductivity.

In difference methods, the resistance of thin well-conducting ion-exchange membranes is defined as a small difference between two large values, and therefore, with a significant error that increases with the dilution of the solution [44,45,46,47,48].

The disadvantages of this method are electroosmotic flow and concentration polarization of membranes, which cannot be avoided even with constant washing of electrodes with an equilibrium solution, and a number of other factors (osmotic transfer of water, diffusion, convection, electromigration) that cannot be eliminated, should be avoided and strictly considered.

The main disadvantage of the method is due to the large error associated with the fact that the membrane resistance is found as the difference between two quantities of the same order. This error is especially pronounced in studies of well-conducting membranes in the region of dilute solutions. This drawback can be partially overcome by the differential difference method, which makes it possible to obtain the concentration dependence of the electrical conductivity of membranes on alternating current in the concentration range of equilibrium solutions of 0.003–0.2 mol/L with an error of no more than 2%. At the same time, the method retains the advantages inherent in this group of methods: the absence of membrane deformation, as well as distortion of the measured value by the transition resistance of the electrode-solution interface or by the surface conductivity of the adhered film of the solution.

An important advantage of this method in comparison with contact methods is the ability to equilibrate the membrane with solutions of different concentrations without disassembling the cell. This makes it possible to quickly obtain the dependence of the specific electrical conductivity on the concentration of the solution for one membrane sample.

One of the varieties of the difference method for measuring the transverse resistance of membranes is the probe difference method, in which four electrodes are used for measurements, similar to the probe method of the strip. The main feature of this method is that, in addition to polarizing electrodes, probe measuring electrodes are located near the membrane surface or in direct contact with it. The use of silver chloride or calomel electrodes makes it possible to measure the electrical resistance by direct current. Alternating current measurements are possible using platinum probe electrodes.

Such a formulation of the problem is also possible when the measurement of the electrical resistance of the membranes is carried out both in direct and in alternating currents. In this case, the measuring probe electrodes represent the spouts of the capillaries of the calomel electrodes brought close to the surface of the membrane, into which the platinum probes are inserted so that their ends coincide with the ends of the capillaries. A feature of the technique for simultaneous measurement of the membrane resistance at direct and alternating currents is the use of grounding of the measuring circuit in the thickness of the electrolyte solution near the membrane using a platinum wire loop to reduce pickup [48].

To measure the specific electrical conductivity, a contact-difference method developed by V.A. Shaposhnik et al. consists of measuring the impedances of two and one membranes in a cell with platinum electrodes and finding their vector difference, which was considered as the true electrical resistance of the membrane. The advantage of the contact-difference method is the absence in the true electrical resistance of the membrane of the values of the electrical resistance of the solution between the electrode and the membrane, as in the contact method, and the possibility of determining the true value of the electrical resistance of the membrane by the difference of two close values, in contrast to the difference method [48].

In [49], the electrical conductivity was measured using the contact-difference method. It consists of the fact that first, one membrane is placed between the electrodes and measurements are taken, which can be considered as a result of the contact method. Then the electrical resistance of the two membranes is measured and the difference in the electrical resistance of the two and one membranes is determined. The contact-difference method has advantages in measuring the electrical resistance in solutions of low concentrations when there is no Donnan-adsorbed electrolyte in the membrane and the true properties of counterions and fixed ions are manifested. The authors measured the electrical resistance of the membrane using low-frequency impedance meters TESLA BM 507 and high-frequency 3535 LCR HITESTER HIOKI E.E. Impedance spectroscopy is used to study transport processes in complex systems, especially in heterogeneous materials [50,51]

The impedances of cation-exchange and anion-exchange membranes of domestic brands have been measured in a wide range of alternating current frequencies, which make it possible to turn the capacitive component to zero. This made it possible to obtain active electrical resistances of membranes by contact and contact-difference methods and establish their significant difference and give preference to the contact-difference method [52,53,54].

In this paper [55] by the method of impedance spectroscopy, the electrochemical characteristics of the heterogeneous ion-exchange membranes MK-40 (H^+^, Na^+^, K^+^, NH_4_^+^—forms) and MA-41 (Cl^−^ and NO_3_^−^ forms) in the range of the alternating current frequencies 100 KHz–20 MHz are researched. The comparison of the contact and contact-differential ways of measuring membrane impedance is performed. It is shown that in the case of impedance contact measuring of the sample, the borders “electrode/membrane” influence the electrochemical impedance system spectrum greatly. In connection with this, the contact-differential variant of the experimental procedure in the impedance measuring of the membrane system is preferable. The interpretation of the received electrochemical impedance spectra in terms of the composite material conductivity is given [56,57]. Based on the method of equivalent circuits it is suggested to represent the impedance of the heterogeneous ion-exchange membrane as a sum of pure resistance (resistance of ion-exchanger particles), sequentially connected with the impedance of the dielectric layers (resistance and capacity of polyethylene and dissolvent) [58].

This study [59,60,61,62] examines ionic conductivity measurements based on the experimental technique in which a membrane directly contacts the electrodes. In the through-plane configuration of this technique, external resistances due to the interfacial region between the membrane and electrodes contribute significantly to the total measured resistance, particularly for thin membranes equilibrated with dilute salt solutions. A non-invasive approach to account for such external resistances based on performing the measurements with membranes having different thicknesses is presented. The total resistance of the electrochemical cell containing membranes with different thicknesses was measured via electrochemical impedance spectroscopy, and the results were extrapolated to zero thickness to determine the magnitude of the external resistances [63,64].

A new method for determining the electrical conductivity of ion-exchange membranes was implemented with four commercial membranes (AMX, CMX, MK-40 and MA-41). It is based on lateral resistance measurements without direct contact between electrodes and membranes [31,65,66,67]. The cell configuration made it possible to determine the membrane conductivity over a wide range of electrolyte concentrations (measurements were carried out in the range 1 × 10^−5^–5 × 10^−1^ M). The structural parameters of the different membranes were inferred from AC conductivities and the microheterogeneous model. They were found in good agreement with literature results obtained by normal measurements, thus confirming the reliability of the proposed method. The main advantage of this method is the possibility to characterize ion-exchange membranes even at low salt concentration unlike usual non-contact methods based on normal measurements [65,66,67,68].

In the work of Filipov [68], the electroosmotic permeability and electrical conductivity of an ion-exchange membrane were studied. The membrane was considered as an ordered set of porous spherical charged particles placed in spherical shells filled with a binary electrolyte solution. It was shown that with an increase in the electrolyte concentration, the specific electrical conductivity of the cation-exchange membrane can monotonically increase in different ways, i.e., with or without an inflection point on the graph.

Accurate measurements of membrane ionic conductivity are important for advancing the fundamental understanding of electric field-driven ion transport in ion-exchange membranes. There is no standardized technique for performing such measurements on membranes equilibrated with salt solutions, despite a longstanding interest in this topic. As a result, discrepancies between reported ionic conductivity values for common commercial membranes are often found in the open literature.

The purpose of the work is to determine the electrical conductivity of industrial ion-exchange membranes MK-40 and MA-40, as well as their basis—granules of a bulk layer of industrial ion exchangers KU-2-8 and EDE-10p, by differential and modified contact methods in electrolyte solutions NaCl, MgCl_2_, Na_2_SO_4_ and BaCl_2_ and the development of a new methodology that will give the values that are closest to the true ones; determination of the dependence of electrical membrane conductivity depending on the type of counterion and concentration equilibrium solution and granules of a bulk layer of ion exchangers on the volume fraction of a dry ion exchanger with different degrees of compaction.

## 2. Materials and Methods

### 2.1. Research Objects

The objects of study were ion-exchange resins of Russian production (IP Shchekinoazot LLC, Tulskaya Oblast, Russia): cation exchanger KU-2-8 and anion exchanger EDE-10p and heterogeneous ion-exchange membranes obtained on their basis: cation-exchange membrane MK-40 and anion-exchange membrane MA-40.

To determine the electrical conductivity of industrial ion-exchange membranes MK-40 and MA-40, as well as their basis granules of a bulk layer of industrial ion exchangers KU-2-8 and EDE-10p, by differential and modified contact methods in electrolyte solutions NaCl, MgCl_2_, Na_2_SO_4_ and BaCl_2_ and the development of a new methodology that will give the values that are closest to the true ones; determination of the dependence of electrical membrane conductivity depending on the type of counterion and concentration equilibrium solution and granules of a bulk layer of ion exchangers on the volume fraction of a dry ion exchanger with different degrees of compaction.

Pressed diaphragms compacted under pressure layer of ion exchanger.

**Figure 1:**
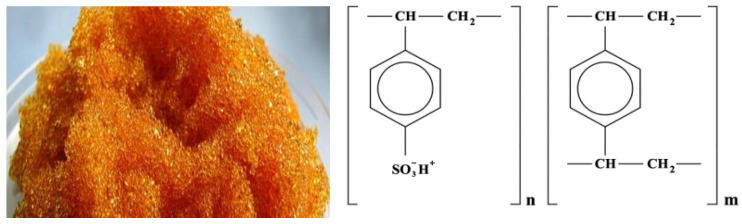
Cation exchanger KU-2-8.

Monofunctional strongly acidic cation exchanger KU-2-8 (GOST 20298—74) is obtained by sulfonation of granular styrene copolymer with 8% divinylbenzene. The proposed structure of the elementary link (Figure 1):

- The functional group is a sulfo group;
- The matrix is styrene-divinylbenzene;
- The structure is gel;
- Particle size 0.4–0.55 mm;
- Moisture content 48–58%;
- Full exchange capacity 4.9–5.1 mmol/g dry exchanger or 2.0 mg·mol/cm^3^;
- Specific volume, no more in H^+^ form 2.7 cm^3^/g.

Differences in high chemical resistance to alkalis, acids, oxidants. It possesses high mechanical strength and osmotic stability. Resistance to high (110–120 °C) temperatures. The range of working pH values is 1–14.

Application: For softening and demineralizing water in water treatment; For the separation and isolation of non-ferrous and rare metals in hydrometallurgy; Wastewater treatment; For the separation and purification of various substances in the chemical industry; In catalytic processes.

**Figure 2:**
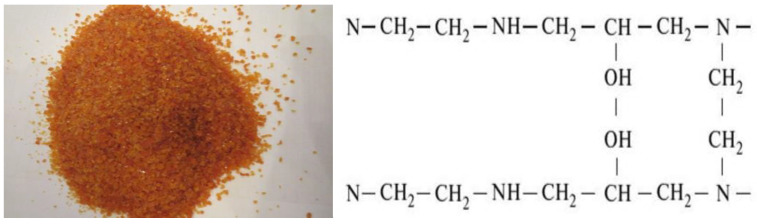
Anion exchanger EDE-10p.

Anion exchanger EDE-10p (GOST 20301-74) is a polyfunctional anion exchanger containing secondary (NH-) and tertiary (-N-) amino groups of the aliphatic series and about 20% of groups of quaternary ammonium bases (Figure 2). The proposed structure of the elementary link:

Anionite is obtained by polycondensation of polyethylenepolyamines with epichlorohydrin, it is produced in Cl^−^:

- The functional group is a NR_3_^+^, =NH, =N
- The structure is a gel
- Particle size 0.4–2.0 mm
- Full statistical exchange capacity, not less than 2.3 mmol/cm^3^;
- Dynamic exchange capacity, m∙mol/m^3^, not less than 1000;
- The volume fraction of the working fraction, is not less than 92%;
- The mass fraction of moisture is no more than 5%;
- The specific volume, in OH^−^ form is 3.4 ± 0.2 cm^3^/g.

Application: The product is used for water treatment, in the production of ion exchange membranes, in the medical and food industries, for the purification of ethyl alcohol from acid impurities and aldehydes.

**Figure 3:**
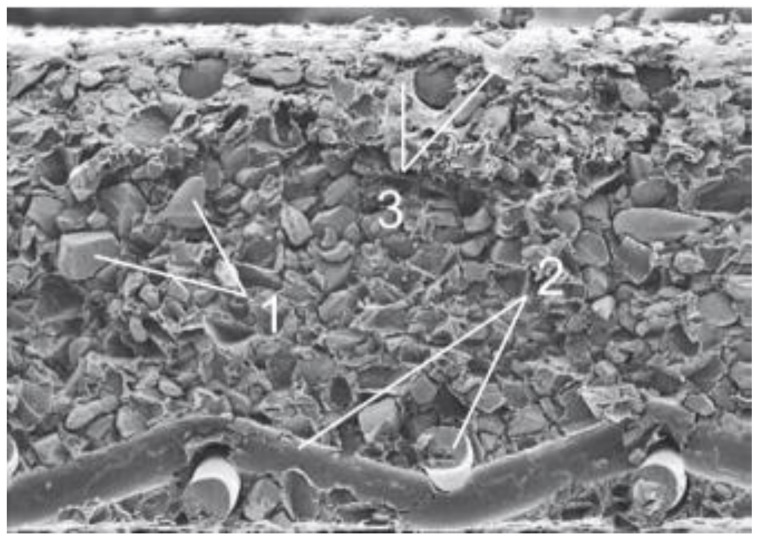
Image of a section of the MA-40 membrane obtained using scanning electron microscopy [37]: 1—particles of anion-exchange resin, 2—reinforcing mesh, 3—polyethylene.

The MK-40 cation exchanger based on the KU-2-8 cation exchanger and the MA-40 anion exchanger, the base, the EDE-10p anion exchanger, is commercially produced (SHEKINOAZOT. LTD, Tulskaya Oblast, Russia) by mixing rather large particles of crushed ion exchanger (5–50 µm) and an inert binding agent, for example, polyethylene particles, which are applied to an inert nylon or nylon reinforcing mesh followed by hot pressing (Figure 3). The content of polyethylene as a binder in the MK-40 and MA-40 membranes is 60–65%.

The particles of the ion exchanger do not melt and do not mix with polyethylene, which, after heating, melts and fills the space between the particles and flows out onto the surface, as a result of which most of the surface of the heterogeneous ion-exchange membrane is non-conductive. In connection with this, the surface of heterogeneous ion-exchange membranes is distinguished by a sharp gradient of physical and chemical properties in the normal and tangential directions [38,39,40,41].

- Heterogeneous membranes have little water permeability.
- For membranes MK-40 and MA-40 at 18 °C and a pressure of 37.2 nPa in distilled water, it is (1¸2) × 10^−12^ g/(cm^2^ × s).
- The diffusion permeability of these membranes is also low: 6.2 × 10^−8^ g/(cm^2^ × s) in 1 n. NaCl at 20 °C.
- Osmotic permeability of MK-40 and MA-40 membranes in 1 N. NaCl at 20 °C is 0.2 and 0.1 L/(m^2^ × h), respectively.
- Functional groups: -SO_3_H (MK-40), -NH_2_ ^+^, = NH, = N (MA-40);
- Moisture content, %, no more than 40 (MK-40, MA-40);
- The content of the ion exchanger, % KU-2-8-65, EDE-10p-55;
- Transfer number in 0.01–0.2 N NaCl fraction, not less than 0.98 (MK-40), 0.94 (MA-40).

The complex structure of heterogeneous ion-exchange membranes determines their special conductive characteristics. In addition to the selective transfer of ions through ion exchanger particles, diffusion transfer of electrolyte through the pores formed in the membranes during production can occur in such membranes. In the works of NP Berezina’s group [42] it was found that for the heterogeneous ion-exchange membrane MK-40, the sizes of most of the pores correspond to 10 nm and 1000 nm. Smaller pores are localized inside the ion exchanger, and larger ones at the interface of dissimilar phases (particles of ion exchange resin, polyethylene, reinforcing mesh) in the volume and on the surface of the membrane. Membranes are used in installations of various directions: demineralization of whey, refining sugar syrups, stabilization of wine raw materials, demineralization and deionization of water, wastewater treatment with the release of valuable elements, deep cleaning of biological and medical products, disinfection of radioactive wastewater, the concentration of wastewater containing valuable components, and water preparation for heat power engineering.

#### Preparation of Research Objects

The membranes were prepared for the study according to the standard method: to remove the oil film, the membrane surface was wiped with a swab with CCl_4_, then the membranes were immersed in ethyl alcohol and kept in it for 6 h. After alcohol treatment, the membranes were immersed in a saturated NaCl solution and then washed with distilled water. Next, the membranes were treated alternately with solutions of 10% HCl and 10% NaOH, after which they were converted into H^+^ and OH^−^ forms of 1M HCl and NaOH for two days with repeated solution changes. After washing with distilled water until the wash water was neutral, the membranes were converted into Na^+^ and Cl^−^ forms, respectively, by multiple treatments with 1N NaCl solution.

Ion exchanger KU-2-8 was treated with 4% HCl, washed with water, then converted into Na^+^ form with 3% NaOH and again washed with water until a neutral pH was reached. EDE-10p, in order to avoid cracking of the granules, was soaked for swelling for 2–3 h in three volumes of 25% NaCl. Then it was washed with water and converted into the Cl^−^ form with 4% HCl and again washed with ~5 volumes of distilled water (to avoid hydrolysis). Before the experiments, membranes and ion exchangers were brought into equilibrium with the test solutions.

### 2.2. Measurement of Electrical Conductivity of a Bulk Layer of Ion Exchangers

The measurement of the electrical conductivity of the ion exchanger granules placed in an equilibrium solution, depending on the compaction, was carried out in a cell, which consisted of two blocks (1) made of plexiglass and glued in such a way that a narrow slotted channel with a height of 7.5 cm and width 1 cm was made. Platinum electrodes (2) were glued into the channel walls, which were connected to the measuring circuit with insulated leads (4) made of copper wire by means of a mercury contact. The measurements were carried out in a cell thermostated in an air thermostat (with an accuracy of 0.10 C) using an R-5021 AC bridge. The measurement error did not exceed ±1%. The determination of the electrical conductivity of the bulk layer of ion exchangers was carried out depending on the volume fraction of the ion exchanger P, achieved at various degrees of compaction, up to the maximum at a pressure of about 100 atm. Compaction was carried out by compressing the piston (3) in a vice, which moved along the channel at a strictly defined fixed distance corresponding to the height of the ion-exchange column h. The volume fraction of dry ion exchanger P, corresponding to different degrees of filling, was calculated using the formula:

P = h_d_0__ P__0__/h_d_ (1)

where:

- h_d_0__, P_0_ respectively the height and volume fraction of the ion exchanger at the initial moment before compression;
- h_d_ is the height of the bulk layer of the ion exchanger corresponding to the degrees of filling for which was determined P.
- The electrical conductivity of the bulk layer of ion exchangers was measured
- depending on the concentration of the equilibrium solution.

### 2.3. Measurement of Electrical Conductivity of Membranes

The measurement of the electrical conductivity of the membranes was carried out by two methods: the well-known difference method and the modified contact method, which consists in measuring the electrical conductivity of moisture-saturated membranes, previously equilibrated with the test solutions, in saturated vapors of the test solutions.

#### 2.3.1. Measurement of the Conductivity of Membranes by the Difference Method

**Figure 4:**
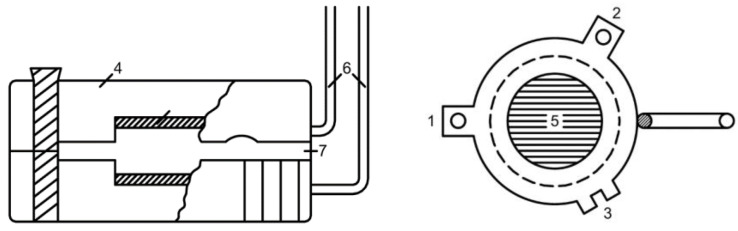
Cell for measuring the electrical conductivity of ion exchange membranes by the difference method. In the figure: 1—disc, 2, 3—protrusions, 4—disc material—plexiglass, 5—platinum electrodes, 6—insulated bushings, 7—gap between the electrodes.

To measure the electrical conductivity of the membranes by the difference method, a slotted cell (Figure 4) was used, which consisted of two discs (1), 36 mm in diameter, made of plexiglass (4), fastened on the protrusions (2, 3) with brass bolts. To maintain a strictly fixed distance, fluoroplastic plates of a certain thickness were placed between the disks. On the inner side of the discs, round platinum electrodes (5) with an open surface diameter of 15 mm were glued in, and deepened with respect to the planes forming the slit. The membrane was placed in the gap (7) between the electrodes. Disk electrodes were connected to the measuring unit with insulated bushings (6). The measurements were carried out in a thermostated cell with alternating current at a frequency of 800 Hz. The measurement error did not exceed ±1%. The resistance of the cell was measured in a solution without a membrane (R_v_) and with a membrane (R). Each operation was repeated 5–10 times and then the difference between these values ∆R = R − R_v_ was calculated.

The membrane resistance was determined from ∆R, Rm = ∆R + R_h_, where Rh is the resistance of the solution layer equivalent to the membrane in area and thickness, Rh = R_v_∙d/a, where d is the membrane thickness (determined by the micrometric method), a is the distance between the electrodes (a = c_v_·S, where c_v_ is the cell resistance constant, S is the area of the electrodes). The specific conductivity of the membrane (${ǣ}_{m}$) was calculated by the formula:$\;{ǣ}_{m}$ = C_h_^0^/R_m,_ where C_h_^0^ = ӕ_v_^0^∙R_h_^0^ (ӕ_v_^0^ is the conductivity of the control solution).

The dependences of the specific electrical conductivity of solutions (ӕ_v_) and membranes (${ǣ}_{m}$) on the concentration of the equilibrium solution were determined. Disks with an area of 10 cm^2^ were cut from a sheet of the original membrane, processed according to the generally accepted technique; for work, disks were strictly selected, the heterogeneity of which in thickness did not exceed 0.05–0.06 cm. The cell was thermostated in an ultrathermostat with an accuracy of ±0.1 °C. Measurements (${ǣ}_{m}$) were carried out in all studied solutions.

#### 2.3.2. Measurement of Conductivity of Membranes by Contact Method

**Figure 5:**
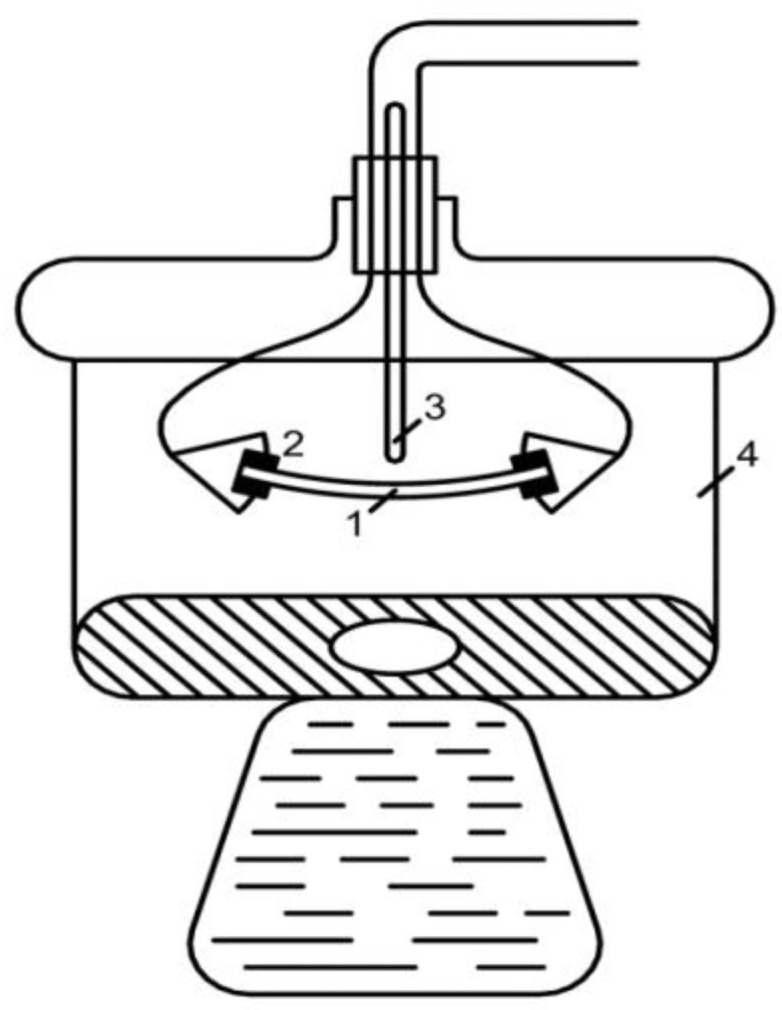
Cell for measuring the electrical conductivity of ion exchange membranes by the contact method. In the figure: 1—strip of membrane, 2—narrow platinum electrodes, 3—thermometer, 4—thermostated closed desiccator.

The measurement of membrane resistance (R_m_) by the contact method (Figure 5) was carried out on strips of membranes (1) 10 cm long and l cm wide using two pairs of narrow platinum electrodes (2), one pair of which was pressed on both sides to the end of the strip, the other could move along the strip at strictly fixed distances, l, from the first pair. 3-thermometer, 4-thermostated closed desiccator.

The solution film was removed from the membrane surface with filter paper. The measurements were carried out in a thermostated closed desiccator in an atmosphere of saturated water vapor. The temperature was recorded with an accuracy of ±0.1 °C. The Rm values measured on alternating current did not change in all experiments for several hours. The deviations of Rm from the mean values for five parallel samples did not exceed 5% and were associated with differences in the thickness of the studied membranes (0.5–0.6 mm).

The obtained dependences R_m_ − l in all cases were linear; extrapolation to l = 0 gave a segment on the ordinate axis corresponding to the ballast resistance R^/^ which was subtracted from the total measured resistance R:

(2) $${ǣ}_{m}=\frac{l}{\left(R-R^{\prime }\right)\cdot S}$$

where S is the cross-sectional area of the sample.

**Figure 6:**
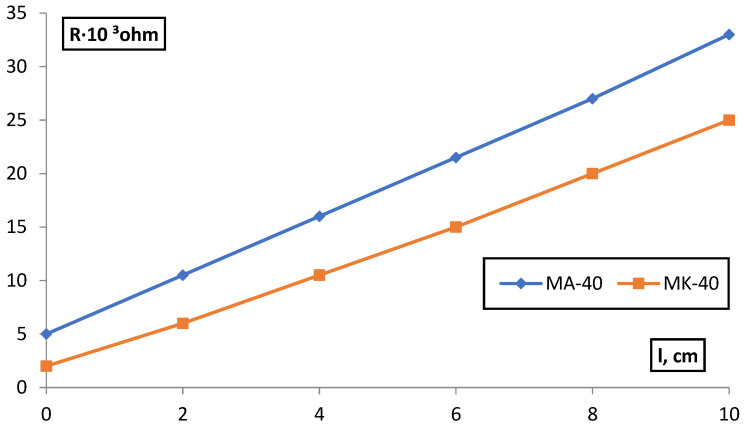
Dependence of the resistance of membranes in equilibrium with 0.1 M NaCl, on the distance between the electrodes.

The R^/^-value was determined for each electrolyte concentration. Figure 6 shows an example of R^/^definition.

The specific electrical conductivity of the solution inside the membrane, both in the difference and in the contact method, was calculated by the formula:

(3) $${ǣ}_{l}={C_{m}}^{0}\;/R_{m}$$

where C_m_^0^ = ӕ_v_^0^·R_m_^0^.

The difference between the values of ǣ_l_ and ǣ_m_ is determined by the resistance of the non-conducting skeleton and is characterized by the coefficient of structural resistance β, which shows how many times the resistance of the uncharged membrane is greater than the resistance of the equivalent layer of the solution β = C_m_^0^/C_h_^0^.

For membranes, the usual method of determination in 0.1 M KCl is inapplicable, since C^−^ ≠ C_0_. Therefore, 0.1 M BaCl_2_ solutions for MK-40 and 0.01M NaOH for MA-40 were used as control solutions for determining C_m_^0^ and C_h_^0^, assuming complete screening of fixed charges in these systems.

**Figure 7:**
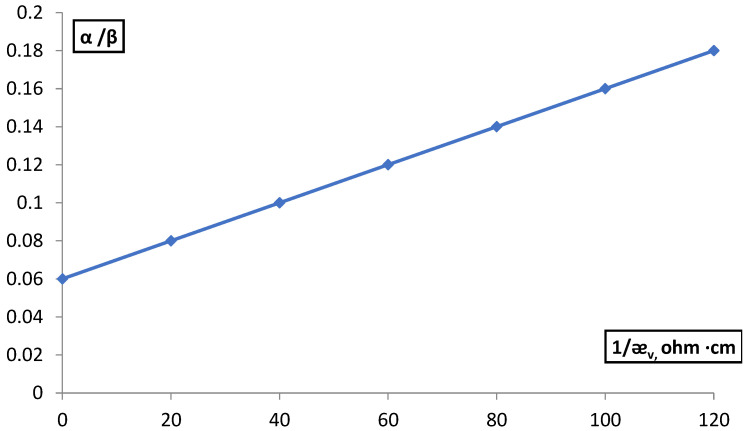
Dependence of the α/β ratio for the MK-40 membrane on the value of the reverse conductivity of BaCl_2_ solutions.

To determine β, the concentration dependence of the electrical conductivity of the membranes was investigated, determined by the difference method in BaCl_2_ and NaOH solutions in the concentration range 1∙10^−1^–4∙10^−1^ M and by extrapolating the direct dependence of α/β on 1/ӕ_v_ (Figure 7).

For MK-40 membranes, the β value was 16.1 and for MA-40 β = 12.5 (in the case of the difference method) and 6.7 and 11.65 in the case of the contact method. The contribution to the conductivity of counter ions was taken into account by the efficiency coefficient α, which shows how many times the electrical conductivity of the solution in the pores of the membrane exceeds the electrical conductivity of the free solution, α = ǣ_l_/ӕ_v_.

## 3. Result and Discussion

To explain the concentration dependences of the electrical conductivity of membranes, a microheterogeneous model was used [69] According to this model, the entire volume of the membrane is divided into two components. This is the volume of an electrically neutral solution, which is identical to the external solution and is called the intergel phase. Such a solution fills the central part of clusters and channels, as well as cracks, cavities, and other defects of a homogeneous structure. The gel phase unites the regions of the charged matrix containing micropores, in which mobile counterions and co-ions compensate for the matrix charge.

**Figure 8:**
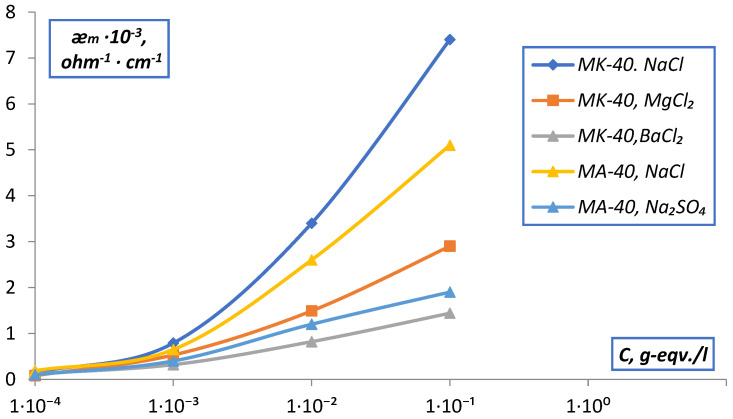
Dependence of the specific electrical conductivity (${ǣ}_{m}$) of membranes measured difference method on the concentration of solutions.

The measurement of the electrical conductivity of the membranes was carried out by two methods: difference and contact methods. The conducted studies of the dependence of the electrical conductivity of MK-40 and MA-40 membranes on the concentration of solutions by the difference method (Figure 8) showed that the values of ${ǣ}_{m}$ for singly charged electrolytes are higher than for doubly charged ones, which confirms the specificity of the interaction of doubly charged ions with the membrane. The values of ${ǣ}_{m}$ for all electrolytes sharply decrease when going from 10^−1^ to 10^−4^ N solutions, which contradicts the accepted concepts for homogeneous ion exchangers, according to which the value of ${ǣ}_{m}$ for ion-exchange membranes is determined by a high concentration of counter ions in the membrane, which should practically not change with dilution (in accordance with the Donnan equilibrium. The reason is the electrically neutral solution contained in the central parts of large pores, and identical to the external solution. In the range of low concentrations of the external solution, the low specific electrical conductivity of the solution in the intergel gaps determines the low conductivity of the membrane as a whole. As the concentration of the solution increases, the conductivity of the intergel gaps increases, and they no longer limit the conductivity of the membranes.

Analysis of the data obtained (Figure 8) shows that in the region of diluted solutions, the conductivity of all membranes rapidly decreases with the dilution of the solution. According to the microheterogeneous model, the reason is the electrically neutral solution contained in the central parts of large pores, and identical to the external solution. In the area of small concentrations of the external solution low specific conductivity of the solution in intergel gaps causes a low conductivity of the membrane as a whole. With an increase in the concentration of the solution, the conductivity of the intergel spaces increases, and they cease to limit membrane conductivity.

However, taking into account that the membranes under study are heterogeneous systems and believing that one of the reasons for the abnormal behavior of membranes may be a complex configuration of current paths in such real heterogeneous membranes due to their structure, it was of interest to study the concentration dependence of electrical conductivity on the initial ion exchange resins.

The dependence of the electrical conductivity of ion-exchange diaphragms ǣ_d_ (volumetric layer of an ion-exchanger in solution) from ion-exchange resins KU-2 and EDE-10p on the volume fraction of the ion exchanger P (calculated as a dry ion exchanger) was determined.

**Figure 9:**
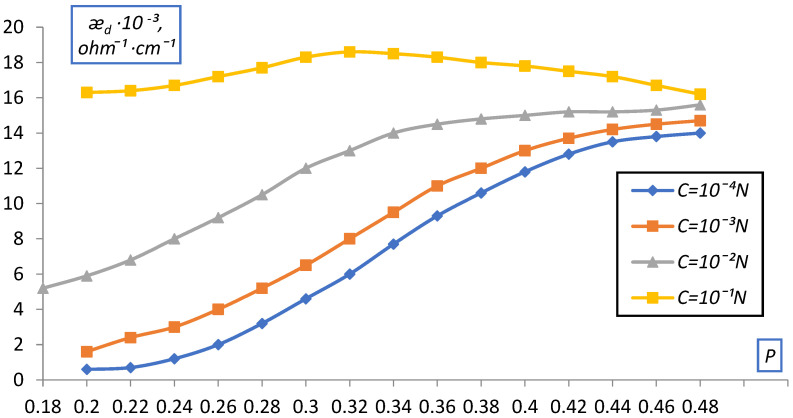
Dependence of the specific electrical conductivity of the bulk layer of the KU-2-8 cation exchanger on the volume fraction of dry ion exchanger in NaCl solution.

**Figure 10:**
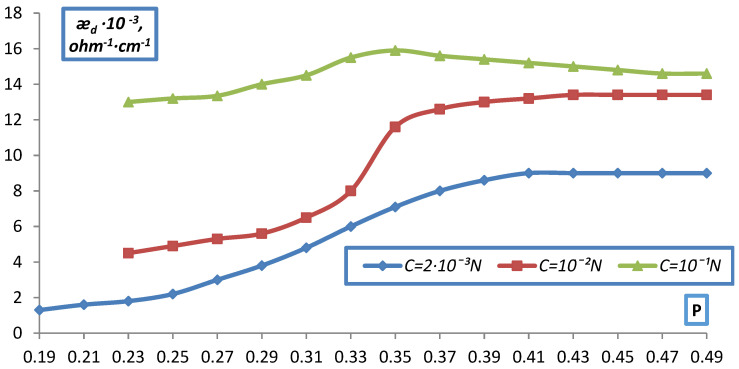
Dependence of the specific electrical conductivity of the bulk layer of the EDE-10p anion-exchanger on the volumetric fraction of dry ion exchanger in NaCl solution.

The fraction of the ion exchanger varied from 0.2, which corresponds to free packing of the ion exchanger grains in the solution, to the maximum (P~0.4), which was achieved at a pressure of about 100 atm and corresponded to the water content in the ion exchanger from vapor P/P_0_ = 1. The data obtained are shown in Figure 9 and Figure 10 as the dependence of æ_d_ on the volume fraction of the ion exchanger P for KU-2 and EDE-10P in NaCl solutions of various concentrations.

As can be seen from the data presented, as the ion exchanger grains become denser, the curves for different C approach each other and the dependence of d on C is quite significant in the uncompacted layer (P = 0.2) and practically disappears at large compaction (P > 0.4).

This, obviously, can be explained by the squeezing out, by compaction, of the solution from the gaps between the grains, which leads to an increase in the contact between them (ǣ_d_ grows as compaction) and at maximum compaction, that is, almost complete absence of solution interlayers between the grains, ǣ_d_ corresponds the ion exchanger itself, possibly even with a somewhat more compacted structure (a decrease in the proportion of gel gaps), which was confirmed by data on the moisture content of the ion exchangers (a decrease in the moisture content in comparison with the initial ion exchanger).

**Figure 11:**
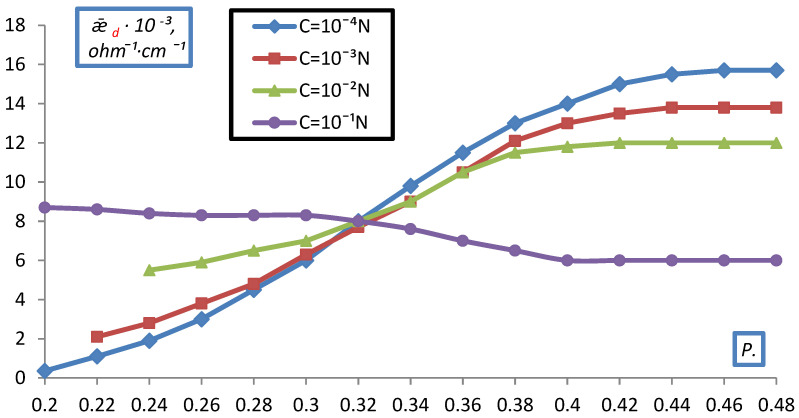
Dependence of the specific electrical conductivity of the bulk layer of the KU-2-8 cation exchanger on the volume fraction of dry ion exchanger in a MgCl_2_ solution.

**Figure 12:**
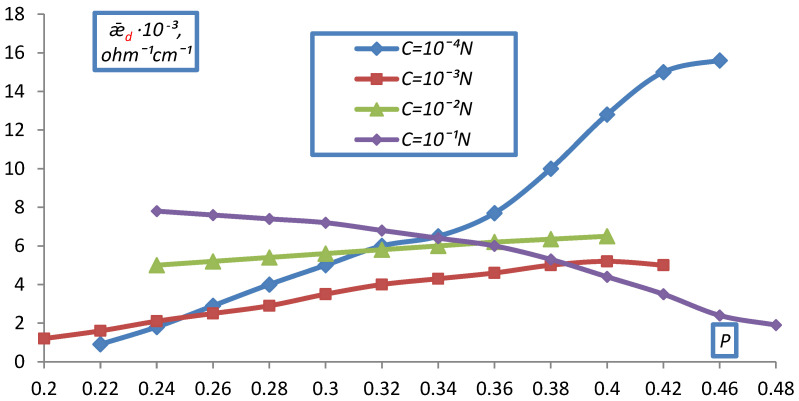
Dependence of the specific electrical conductivity of the bulk layer of the KU-2-8 cation exchanger on the volume fraction of dry ion exchanger in a BaCl_2_ solution.

**Figure 13:**
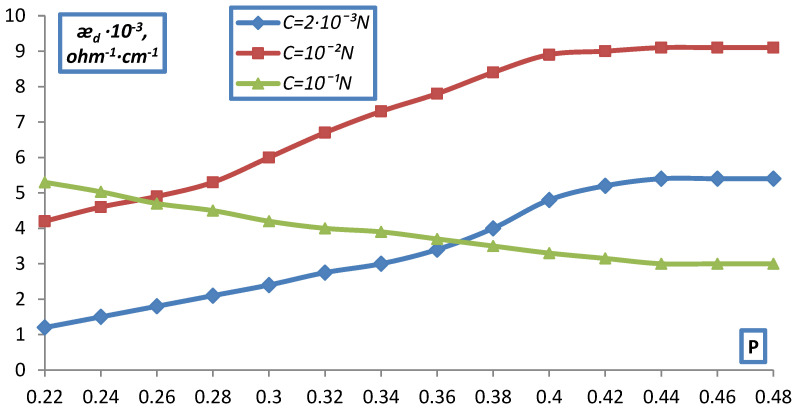
Dependence of the specific electrical conductivity of the bulk layer of the EDE-10p cation exchanger on the volume fraction of dry ion exchanger in a Na_2_SO_4_ solution.

A similar picture of the dependence of ǣ_d_ on C was observed for doubly charged ions in dilute solutions (Figure 11, Figure 12 and Figure 13).

However, in 10^−1^ N solutions of MgCl_2_, BaCl_2_, Na_2_SO_4_, a decrease in ǣ_d_ was observed with compaction, due to the fact that as compaction increases the contribution of conductivity to ǣ_d_ in the form of low-mobile doubly charged ions, which screen fixed ions, crosslink the resin, and the fraction of the electrical conductivity of the solution decreases.

From the electrical conductivity of ion-exchange diaphragms at maximum compression, when their conductivity corresponds to the conductivity of the ion-exchanger, in 0.1 N BaCI_2_ and NaOH, we determined the values of the structural resistance coefficients of the ion-exchanger β. It was found that β = 7.7 for KU-2-8 and 3.12 for EDE-10p.

When comparing the obtained dependencies, it can be seen that for all densities up to the maximum, an increase in electrical conductivity ǣ_d_ is observed, the slower the increase in P. At maximum compaction in NaCl solutions, the value of ǣ_d_ increases insignificantly compared to ǣ_m_ for membranes, and in MgCl_2_ and BaCl_2_ solutions, a drop in the conductivity of the ion exchanger is observed at C > 10^−2^ N. The reason for this is the low conductivity of the resin in the form of a doubly charged ion.

For EDE-10p in Na_2_SO_4_ solution, all curves corresponding to different P pass through a maximum. Here, apparently, the peculiarities of the structure of the polyfunctional ion exchanger EDE-10p are reflected. As seen from obtained data, the electrical conductivity of ion exchange diaphragms, as well as the electrical conductivity of membranes, decreases with the size of the ion, namely ${ǣ}_{{\mathrm{Na}}^{+}}\;>\;{ǣ}_{{\mathrm{Mg}}^{+2}}$ > ${ǣ}_{{\mathrm{Ba}}^{+2}}$ and ${ǣ}_{{\mathrm{Cl}}^{-}}>{ǣ}_{{\mathrm{SO}}_{4}^{-2}}$. In the same direction, the value of the electrical conductivity of the solution in the membrane, ǣ_l_, calculated from the values of ǣ_m_ (according to the formula ǣ_l_ = ǣ_d_∙β at P = P_max_) and the efficiency coefficients α, characterizing the contribution of counter ions to the conductivity of the ion exchanger, decreases.

Thus, from the data presented, it can be seen that in solutions of singly charged electrolytes at the maximum densification of the diaphragms, the conductivity increases relatively little compared with an increase in the concentration of the solution. Hence it follows that the ǣ_m_, C dependence (a change in m by two orders of magnitude) observed when measuring by the difference method can be caused not only by the complexity of the membrane structure but also by errors in determining ǣ_m_ (as the difference of two large quantities); therefore, we have developed a contact method for measuring the electrical conductivity membranes in saturated water vapor.

**Figure 14:**
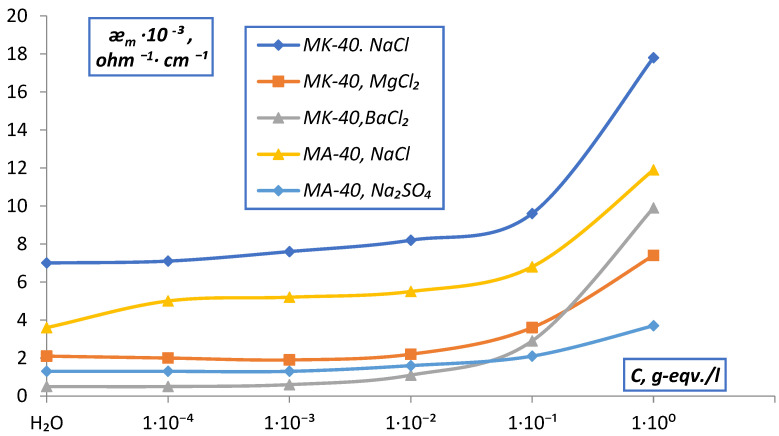
Dependence of the specific electrical conductivity of membranes (ǣ_m_) measured by the contact method on the concentration of solutions.

The results obtained by measuring ǣ_m_ of MK-40 and MA-40 membranes in solutions of simple electrolytes by the contact method are shown in Figure 14.

The obtained values of the electrical conductivity of MK-40 membranes in NaCl solutions show that the values of ǣ_m_, as well as ǣ_d_ at the maximum compaction of the ion exchange diaphragm (P = P_max_), practically do not change in the range from 10^−4^–10^−2^ N and slightly increase (~2 times) in the range of 10^−2^–1 N solutions. This is in accordance with the theory of membrane equilibrium and allows one to explain the dependence ǣ_m_, C without invoking the concept of membrane heterogeneity. A similar picture was observed for the MA-40 membrane and the EDE-10p anionite.

It should also be noted that the dependences of the electrical conductivity of the membranes on the nature of the equilibrium solution agree with the previously obtained sequences for ion exchange diaphragms. The observed series of dependences is explained by the fact that the values of the hydration energy in this series decrease on going from Na^+^ to Ba^+2^, the electrostatic interaction between the fixed ion-counterion increases, and the membrane electrical conductivity decreases. A sharper increase in the electrical conductivity of membranes in BaCl_2_ solutions can be caused by the greater specificity of Ba^+2^ ions to the sulfo groups of fixed ions and the incorporation of Donnan adsorbed electrolyte.

Comparison of the data obtained by contact and difference methods shows that the difference method can only be applied when measuring ǣ_m_ in concentrated solutions, where the determination error is minimal.

In the region of low concentrations of the external solution, the conductivity of the membrane is higher than the conductivity of the equilibrium solution. This is because the concentration of fixed membrane groups is constant. They are associated with an equivalent amount of counterions, the concentration of which in the membrane is higher than the concentration of the same ions in an equilibrium solution. With an increase in the concentration of the solution, the conductivity of the solution grows faster than the conductivity of the membrane, which contains a limited number of counterions. At a certain concentration of the external equilibrium solution, the conductivity of the membrane and the solution are equal [55].

The point of intersection of the concentration dependences of the conductivity of the solution and the membrane is called the point of isoelectric conductivity. The value of electrical conductivity at this point is determined by the conductivity of the gel phase of the membrane and gives an idea of the transport properties of the ion-conducting polymer from which the membrane is made. It was found graphically as the intersection point of the concentration dependences of the specific conductivity of the membrane and the conductivity of the solution [31,66,67].

**Figure 15:**
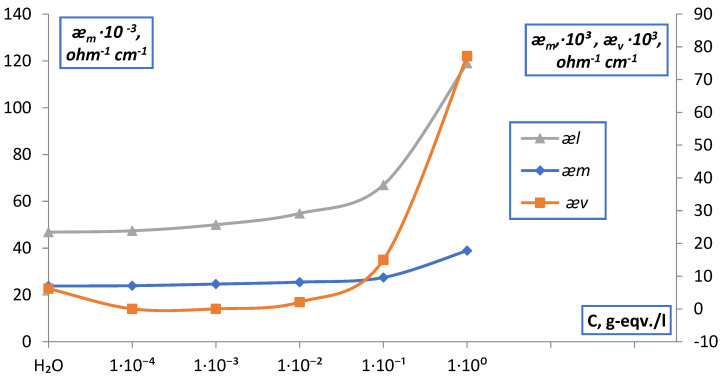
Point of isoconductivity of membrane MK-40 in NaCl solution (contact method).

**Figure 16:**
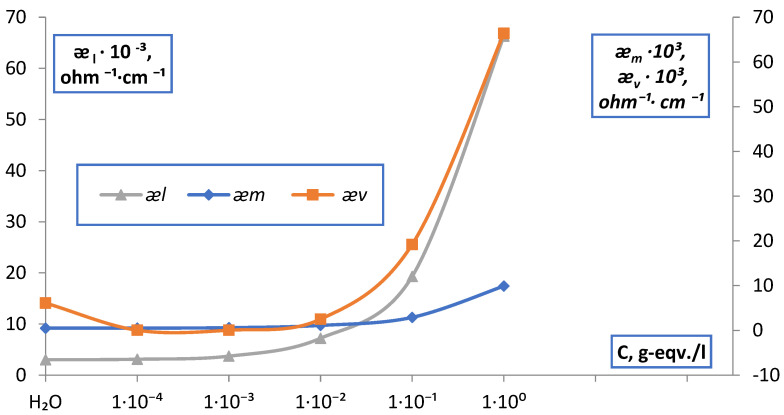
Point of isoconductivity of membrane MK-40 in BaCl_2_ solution (contact method).

**Figure 17:**
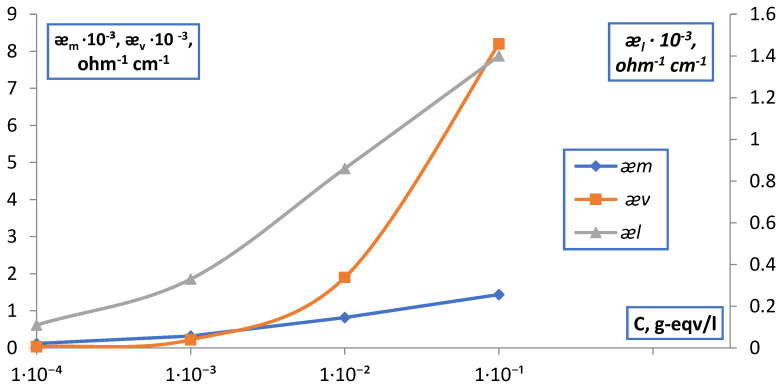
Point of isoconductivity of membrane MK-40 in BaCl_2_ solution (difference method).

For membranes MK-40 and MA-40, in all electrolyte solutions NaCl, MgCl_2_, Na_2_SO_4_, BaCl_2_, points of isoconductivity were found as points of intersection of the curves of the dependences ${ǣ}_{m}\;\mathrm{and}\;{\bar{æ}}_{v}$. The data obtained are shown in Figure 15, Figure 16 and Figure 17 and in Table 1.

Additionally, in solutions of inorganic electrolytes, it was determined moisture content, the volume fraction of dry ion exchanger and density of ion exchanger grains in solutions of inorganic electrolytes. The data are shown in Table 2. The data obtained are in good agreement with the literature data [68,69,70].

From the data obtained, it follows that with an increase in the charge of the ion, the specificity of its interaction with the matrix of the ion exchanger increases and, accordingly, the water content decreases.

The moisture capacity of membranes was determined and its dependence on pH and concentration of solutions was investigated. It was found that the moisture capacity of MK-40 and MA-40 membranes does not depend on pH (in the pH range 3–10) or on the concentration of the solution, in NaCl and MgCl_2_ solutions the moisture capacity MK-40 is 37%, the moisture capacity of MA-40 in NaCl and Na_2_SO_4_ solutions is 40%, which is in good agreement with the literature data [71,72].

## 4. Conclusions

The study of the electrical conductivity of the diaphragms (ǣ_d_) from the grains of the ion exchanger showed that as the diaphragm becomes denser under pressure, the dependence of æ_d_ on the C concentration of the electrolyte disappears in accordance with theoretical concepts. Comparison of the electrical conductivity of the membranes, measured by the difference and contact methods, showed that the difference method gives lower values of ǣ_m_ in the region of low concentrations, while the data obtained by the contact method are in good agreement with the results obtained for pressed diaphragms. The conductivity of the membranes decreases with increasing ion size: ${ǣ}_{{\mathrm{Na}}^{+1}}\;>\;{ǣ}_{{\mathrm{Mg}}^{+2}}$ > ${ǣ}_{{\mathrm{Ba}}^{+2}}$ and ${ǣ}_{{\mathrm{Cl}}^{-}}>{ǣ}_{{\mathrm{SO}}_{4}^{-2}}.$ In the same series, ion mobility decreases. For membranes MK-40 and MA-40, isoconductivity points were found in all electrolyte solutions NaCl, MgCl_2_, Na_2_SO_4_, BaCl_2_. It was found that for MK-40 membranes the value of the structural resistance coefficient β was 16.1 and for MA-40 β = 12.5 (in the case of the difference method) and 6.7 and 11.65 in the case of the contact method.

Measurement of the electrical conductivity of ion-exchange membranes in equilibrium electrolyte solutions is of great importance for the theory of membrane processes, in particular, for practical electrodialysis during desalination and wastewater treatment for calculating the most optimal technological modes of operation of the apparatus.

## Figures and Tables

**Table 1:** Points of isoconductivity of membranes in solutions of simple electrolytes.

C, g-eqv./l					
Electrolyte	Ion Exchanger/Solution	Ion Exchanger-Membrane/Contact Method	Membrane–Solution/Contact Method	Ion Exchanger Membrane/Difference Method	Membrane–Solution/Difference Method
NaCl	0.21	1.5	0.079		0.06
MgCl_2_	0.067	0.15	0.024		0.04
BaCl_2_	0.047	0.15	0.006	0.045	0.001
NaCl	0.16	2.2	0.056		0.03
Na_2_SO_4_	0.047	0.15	0.017	0.148	0.011

**Table 2:** Moisture content, volume fraction of dry ion exchanger and density of ion exchanger grains in solutions of inorganic electrolytes.

Ion Exchanger	Form	Density, g/cm^3^	Moisture Content, ω_0,%_	Volume Fraction of Dry Ion Exchanger P_0_
KU-2-8	Na^+^	1.40	0.6	0.4
	Mg^+2^	1.45	055	0.45
	Ba^+2^	1.73	0.52	0.48
EDE-10p	Cl^−^	1.29	0.52	0.47
	SO_4_^−2^	1.34	0.54	0.46

## Abbreviations

MK-40	industrial cation exchange membrane
MA-40	industrial anion exchange membrane
KU-2-8	industrial cation exchanger
EDE-10p	industrial anion exchanger
ǣ _l_	electrical conductivity solution in the membrane, ohm^−1^∙cm^−1^
ǣ _m_	electrical conductivity of ion exchange membranes, ohm^−1^∙cm^−1^
ǣ _d_	electrical conductivity ion exchange resin, ohm^−1^∙cm^−1^
ӕ_v_	electrical conductivity solution, ohm^−1^∙cm^−1^
β	coefficient of structural resistance
R	resistance, ohm
l	distance between the electrodes, cm.
α	efficiency coefficient
C	concentration, g-eqv./l
P	volume fraction of dry ion exchanger
N	normal concentration, g-eqv./l
